# Biological and technical challenges for implementation of yeast‐based biosensors

**DOI:** 10.1111/1751-7915.14183

**Published:** 2022-11-23

**Authors:** Ehtisham Wahid, Ohiemi Benjamin Ocheja, Enrico Marsili, Cataldo Guaragnella, Nicoletta Guaragnella

**Affiliations:** ^1^ DEI – Department of Electrical and Information Engineering – Politecnico di Bari Bari Italy; ^2^ Department of Biosciences, Biotechnologies and Environment – University of Bari “A. Moro” Bari Italy; ^3^ Nottingham Ningbo China Beacons of Excellence Research and Innovation Institute Ningbo China

## Abstract

Biosensors are low‐cost and low‐maintenance alternatives to conventional analytical techniques for biomedical, industrial and environmental applications. Biosensors based on whole microorganisms can be genetically engineered to attain high sensitivity and specificity for the detection of selected analytes. While bacteria‐based biosensors have been extensively reported, there is a recent interest in yeast‐based biosensors, combining the microbial with the eukaryotic advantages, including possession of specific receptors, stability and high robustness. Here, we describe recently reported yeast‐based biosensors highlighting their biological and technical features together with their status of development, that is, laboratory or prototype. Notably, most yeast‐based biosensors are still in the early developmental stage, with only a few prototypes tested for real applications. Open challenges, including systematic use of advanced molecular and biotechnological tools, bioprospecting, and implementation of yeast‐based biosensors in electrochemical setup, are discussed to find possible solutions for overcoming bottlenecks and promote real‐world application of yeast‐based biosensors.

## Graphical abstract

Yeast‐based biosensors combine the microbial with the eukaryotic advantages, including possession of specific receptors, stability and high robustness. Most yeast‐based biosensors are still in the early developmental stage, with only a few prototypes tested for real applications. Biological, molecular and technical issues are discussed to find possible solutions for overcoming bottlenecks and promote real‐world application of yeast‐based biosensors.

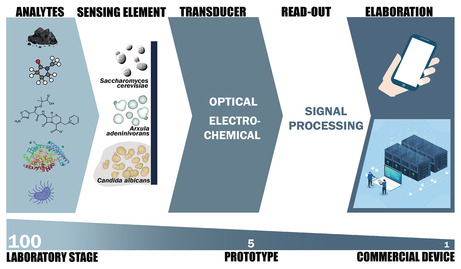

## INTRODUCTION

Biosensors are analytical devices consisting of a biological receptor acting as sensing element and a transducer that converts a biological response into an optical or electrochemical signal for the detection and quantification of a target analyte (Gutiérrez et al., 2015). Due to their small size and low cost, biosensors can be integrated into wide sensor networks (Hart & Martinez, 2006; Phan et al., 2022). Biosensors represent a good alternative to the conventional assay methods requiring complex, offline instruments such as gas chromatography, liquid chromatography, and mass spectrometry. Tested bio‐recognition elements include antibodies, enzymes, receptors, antigens, nucleic acids, organelles, whole cells or tissues, each having specific properties and features for different biosensors' applications. Microorganisms, both prokaryotic (bacteria) and eukaryotic (yeasts and microalgae), have been well studied as biorecognition elements in biosensors, combining the biological receptor and the transducer in one component. In general, interactions with the target analyte results in a functional information expressed by changes in physiological, morphological or biochemical parameters (e.g. cell yield, growth rate, shape, permeability, survival and metabolism). Microbial biosensors (MBs) present several biological, technical and economic advantages over other traditional analytical methods, including indication of bioavailability of analytes, self‐sustaining biorecognition element, user‐friendliness and low cost (Lei et al., 2006; Shin, 2011; Su et al., 2011; Inda et al., 2019; Moraskie et al., 2021). In addition, the application of “omics” technologies, metabolic engineering and in silico modelling methods have significantly improved microbial genetic manipulation, thus offering new prospects for the design of more precise and sensitive MBs.

Most MBs are based on bacterial cells, however a recent interest in yeast‐based biosensors has emerged, especially due to their stability, high robustness and possession of unique eukaryotic receptors. Several yeast species, with *Saccharomyces cerevisiae* as the most prevalent, have been used to develop sensor strains at laboratory scale for the detection of multiple analytes, including endocrine disruptors, trace metal elements, pesticides and fungal pathogens, enabling potential application in the biomedical, industrial and/or environmental field. However, only few prototypes have been tested for real‐world applications. In this review, we described recently developed yeast‐based biosensors highlighting their different features and developmental stage. We focused on the biological aspects, analytical performance, safe deployment, stability and technical issues, considering the gaps limiting the transition from the proof of concept to prototypes as open research challenges. Potential approaches and best practices, such as systematic application of advanced molecular and biotechnological tools, exploitation of genetic and phenotypic biodiversity and implementation of yeast‐based biosensors on electrochemical platforms are proposed as possible solutions.

## YEAST‐BASED BIOSENSORS

**FIGURE 1:**
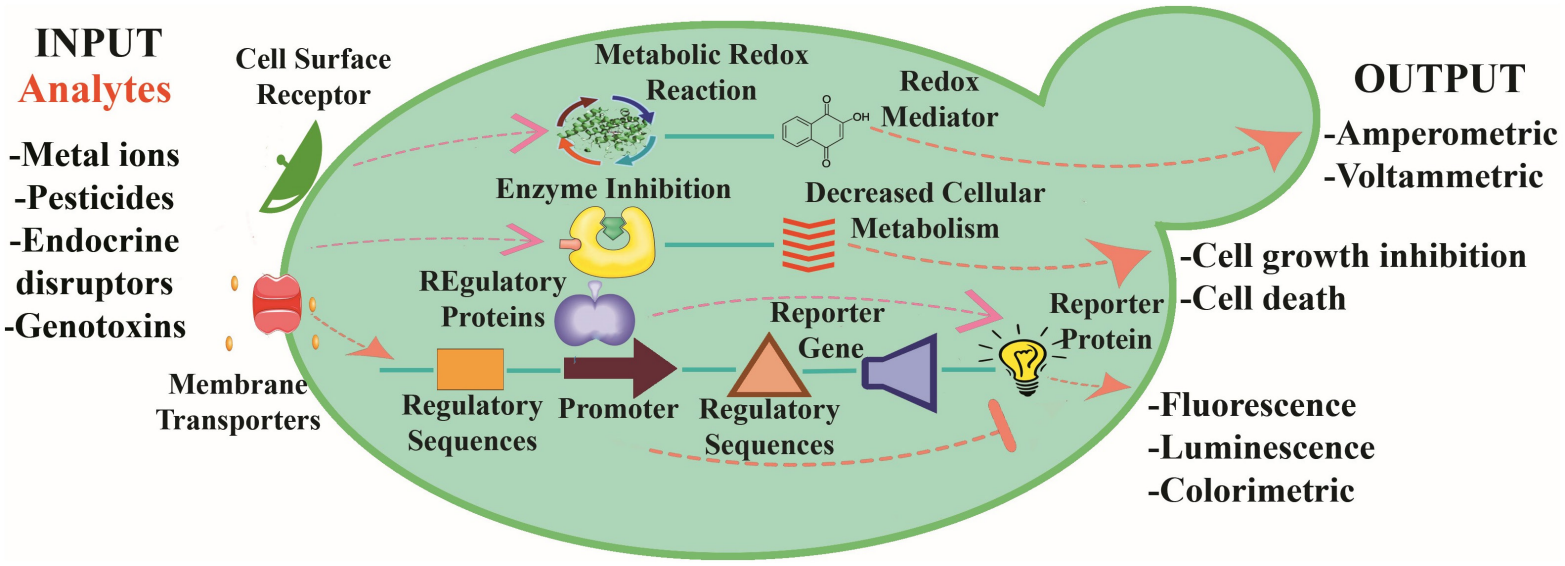
Transduction mechanisms of yeast‐based biosensors. Different yeast species, genetically modified or not, can act as transducers for the sensing and quantification of many analytes. Cell surface receptors and plasma membrane transporters are involved in the first interaction between the cell and the analyte. The output signals can be quantified through: amperometric/voltammetric measurements by means of intracellular metabolic redox reactions and redox mediators; cell growth inhibition and/or cell death by means of inhibition of metabolic regulatory proteins; optical measurements by means of the regulated activity of constitutive or inducible promoters fused to selected reporter genes and related proteins

Yeasts are a suitable platform for biosensor development. Being eukaryotic cells, the separation and compartmentalization of genetic and metabolic events offer the opportunity to precisely control and program gene expression for higher order biological functions. In addition, the knowledge of yeast physiology, biochemistry and genomics, together with the advancements in synthetic biology, make yeast advantageous for genetic manipulation aimed at monitoring and controlling of cellular processes, with high spatial–temporal resolutions (Marsafari et al., 2020). Unlike other in vitro sensors based on non‐viable biological elements, such as enzymes and aptamers, whole‐cells yeast biosensors allow specific detection of compounds in their bioavailable forms without pretreatment and given the less‐demanding growth conditions of yeasts, they are more suitable for on‐site applications and potentially adaptable to portable devices for in situ testing (Adeniran et al., 2015; Jarque et al., 2016; Martin‐Yken, 2020; Roda, Roda, et al., 2011). Yeast cells either possess intrinsic properties to accomplish the sensing and transduction function in their natural state or can be genetically modified, with the latter being the most common choice. In the genetically modified sensor strain, selected regulatory sequences are fused to universal reporter genes, such as *lacZ*, *gfp* or *luc/lux* genes encoding β‐galactosidase, green fluorescent protein (GFP) or firefly/bacterial luciferase, respectively. Their activity can be finely tuned by a direct or indirect interaction with the target analytes and transduced into an optical signal expressed as colorimetric, fluorometric or luminometric changes. The expression of reporter genes can be either constitutive or inducible. In the first case, when the target analyte is present, cell parameters such as growth, survival or metabolic activities are indirectly reflected by the expression of the reporter gene. Yeasts cells can be genetically modified to express heterologous or chimeric proteins to sense the target analyte and generate an output signal, which can be associated to a change in a reporter activity, cell metabolism, growth or viability for quantitative or semi‐quantitative measurement (Figure 1). On the other hand, the design of inducible reporter genes is essentially based on two approaches: either (a) the target compound binds a single regulatory protein causing the inhibition of reporter gene expression (inducible negative regulation), or (b) the target compound binds a receptor protein, located on the plasma membrane or inside the cell, thus activating the expression of the reporter gene (inducible positive regulation). The inducible sensor strains are more specific than the constitutive ones since their activity can be directly modulated by the target analyte and the response is proportional to its concentration. The biosensor sensitivity will be further increased by the intracellular transcriptional and translational cascade mechanism activated by the target analyte (Figure 1).

A variety of compounds have been quantified in liquid and solid samples through yeast‐based biosensors: endocrine disruptors compounds (EDC), trace metal elements, genotoxins and cytotoxins, organic compounds and reactive oxygen species (Table 1). Yeast‐based biosensors have also found application in the assessment of biological oxygen demand (BOD) to monitor water quality (Christwardana et al., 2021; Nie et al., 2015; Osman et al., 2015; Vopálenská et al., 2015). For its marked robustness, genetic tractability and established human safety, *S. cerevisiae* is the most utilized among yeast species for the development of biosensors. However, several other yeast species such as *Hansenula polymorpha*, *Kluyveromyces fragilis*, *Kluyveromyces marxianus*, *Candida tropicalis*, *Pichia pastoris*, and *Arxula adenivorans* have also been proposed as model organisms for biosensor design (Martin‐Yken, 2020).

**TABLE 1:** Representative yeast‐based biosensors developed in the period 2001–2021

Bioreceptor	Target analyte	Output signal	LOD	Status (laboratory/prototype)	Reference
*S. cerevisiae*	Acetic acid	Fluorescence	5 mM	Laboratory	Hahne et al., 2021
*C. tropicalis*	L‐ascorbic acid	Amperometric/voltammetric	62/59 μM	Laboratory	Akyilmaz et al., 2020
*S. cerevisiae*	Oestrogen estradiol 17‐β	Bioluminescence	nanoYESα ‐ 7.1 ± 0.4 nM nano YESβ ‐ 0.38 ± 0.08 nM	Laboratory	Lopreside et al., 2019
*S. cerevisiae*	Estradiol 17‐β	Fluorescence	1 nM	Prototype	Lobsiger et al., 2019
*S. cerevisiae*	Estradiol 17‐β	Bioluminescence	0.08 nM	Laboratory	Cevenini et al., 2018
*S. cerevisiae*	*C. albicans*	Fluorescence/colorimetric	0.01 nM	Prototype	Ostrov et al., 2017
*D. hansenii*	BOD	Amperometric	2.5 mg L^−1^	Laboratory	Zaitseva et al., 2017
*S. cerevisiae*	Genotoxins (MMS, 4‐NQO)	Fluorescence	–	Laboratory	Tian et al., 2017
*P. angusta* *C. curvatus*	Methanol	Amperometric	0.7 μM	Laboratory	Ponamoreva et al., 2015
*S. cerevisiae*	Cu	Colorimetric	1 μM	Laboratory	Vopálenská et al., 2015
*P. angusta*, *A. adeninivorans*, *D. hansenii*	BOD	Amperometric	2.4 mg L^−1^	Laboratory	Yudina et al., 2015
*A. adeninivorans*	Androgens 5α‐dihydrotestosterone	Fluorescence	0.2 nM	Laboratory	Gerlach et al., 2014
*S. cerevisiae*	Cd	Fluorescence	0.2 μM	Laboratory	Matsuura et al., 2013
*S. cerevisiae*	2,4‐DNT	Bioluminescence	–	Laboratory	Fukutani et al., 2012
*A. adeninivorans*	Estrogens estradiol 17‐β	Biochemical/ amperometric	0.02 nM	Prototype	Pham et al., 2012
*S. cerevisiae*	Cu	Bioluminescence	0.5 μM	Laboratory	Roda, Roda et al., 2011
*S. cerevisiae*	Androgens (testosterone)	Bioluminescence	0.5 nM	Laboratory	Roda, Cevenini et al., 2011
*S. cerevisiae*	Cu	Amperometric	0.8 μM	Laboratory	Tag et al., 2007
*S. cerevisiae*	As, Fe, Pb, Cd	Fluorescence	2.5 ppm	Laboratory	Radhika et al., 2005
*S. cerevisiae*	ROS (menadione)	Fluorescence	0.001 μM	Laboratory	Yang et al., 2005
*S. cerevisiae*	Cu	Fluorescence	0.5 μM	Laboratory	Shetty et al., 2004
*S. cerevisiae*	Cu	Amperometric	0.5 mM	Laboratory	Lehmann et al., 2000

Abbreviations: 2,4‐DNT, 2,4‐dinitrotoluene; 4‐NQO, 4‐nitroquinoline‐N‐oxide; MMS, methylmethane sulfonate.

Representative examples of yeast‐based biosensors developed in the last 20 years with their general technical features, specific targets and limit of detection (LOD) are reported in Table 1.

Sensitive optical biosensors for EDC determination (LOD 0.08 nM for 17β‐estradiol) have been developed at a laboratory stage together with two prototypes (Cevenini et al., 2018; Lobsiger et al., 2019; Lopreside et al., 2019; Pham et al., 2012, 2013). In this case, the ability of yeast cells to correctly express transfected vertebrate nuclear receptors and the consequent generation of transgenic yeast strains represents an advantage with respect to bacterial sensors. Numerous studies have demonstrated the efficacy of yeast systems to investigate the presence and also the endocrine activities of pesticides in environmental samples such as wastewater effluents, fish oils, food and soil. In particular, the systems proposed by Cevenini et al. (2018) and Lopreside et al. (2019) represent an upgrade of the widely used yeast oestrogen screening (YES) assays, which employs the NanoLuc luciferase as the reporter protein fused to human oestrogen receptors. In addition, the biosensor of Cevenini et al. (2018) has the possibility for wireless connection to the camera of any smartphone model, which is relevant for on‐site application.

Engineered *S. cerevisiae* cells have been used for biomimetic odour‐sensing system to detect harmful agents, such as 2,4‐dinitrotoluene (2,4‐DNT). Mammalian olfactory receptors coupled to reporter genes, *gfp* or *luc*, were expressed on the yeast cell surface membrane and the signal transduction stimulated from the external ligand through the endogenous yeast G‐protein signalling pathway (Fukutani et al., 2012; Radhika et al., 2007).

Water analyzers based on yeast cells have also been developed for BOD determination for broad applications. Amperometric BOD sensors using the high stress tolerant species *Debaryomyces hansenii* and/or similar co‐cultures, including *Pichia angusta* and *A. adeninivorans*, showed long‐time stability, selectivity and high sensitivity with a LOD of 2.5 mg О_2_/L (Yudina et al., 2015; Zaitseva et al., 2017). A co‐culture‐based biosensor, involving the methylotrophic species *P. angusta* and the oleaginous *Cryptococcus curvatus* has also been developed for the detection of methanol (Ponamoreva et al., 2015).

A fluorescence *S. cerevisiae* sensor has been generated by a genomic integration strategy and validated for the detection of acetic acid in condensation samples from a biogas plant (Hahne et al., 2021). Recently, an acetic acid biosensor based on the *S. cerevisiae* transcription factor Haa1 has been developed for different purposes such as high‐throughput screening of strains producing acetic acid and investigation of acetic acid‐tolerant strain libraries, which are relevant aspects in the development of robust cell factories for conversion of biomass (Mormino et al., 2021).

Another interesting example is the colorimetric yeast‐based biosensor developed by Ostrov et al. (2017) for the detection of the major human, agricultural and food spoilage pathogens, including *Candida albicans*, *Botrytis cinerea* and *Fusarium graminearum*, in complex samples. This modular biosensor, developed in a simple dipstick test, is based on *S. cerevisiae* cells genetically modified to express specific fungal receptors recognizing mating peptides secreted by different pathogenic fungi. Based on similar principles of genetic modifications, a yeast sensor strain in which the endogenous stress‐sensing cAMP‐PKA pathway has been coupled to the mammalian CREBP‐CRE‐stimulated gene expression pathway driving the activity of a GFP reporter protein, has been developed by Radhika et al. (2005) for metal‐ion detection.

The high sensitivity of yeast cells to trace metal elements and the well‐characterized molecular response to metal stress explains why various yeast sensor strains have been developed for trace metal detection. Copper (Cu) has received much attention because of the presence of inducible *CUP1* promoters, which can be easily fused to reporter genes, such as *ade2* for colorimetric, or *lacZ* and *gfp* for fluorometric or luminometric detection (Roda, Cevenini, et al., 2011; Shetty et al., 2004; Tag et al., 2007; Vopálenská et al., 2015). In all cases, the Cu detection activity ranges between 0.5 μM and 500 mM. An electrochemical biosensor based on mixed microbial consortium containing *S. cerevisiae* together with *E. coli* and *B. subtilis* bacterial species has also been developed for both copper (Cu^2+^) and cadmium (Cd^2+^) detection in wastewater (Gao et al., 2016). Recently, a very sensitive yeast‐based biosensor allowing detection of bioavailable copper (10 nM) has been developed by combining a dual‐reporter fluorescent strategy with significant promoter and transactivator engineering (Žunar et al., 2022).

## OPEN RESEARCH CHALLENGES FOR YEAST‐BASED BIOSENSORS

Yeast‐based biosensors offer various advantages for wide sensor networks applications, nevertheless only few prototypes have been realized so far, suggesting apparent shortcomings in the transition from lab‐scale to prototype or full‐scale (Lobsiger et al., 2019; Ostrov et al., 2017; Pham et al., 2012, 2013). Among them, the sensor assembly for operation and stability, its time of response and the different aspects associated to genetic modifications and transduction mechanisms still represent open challenges.

### Safe deployment and shelf life

Cell viability and stability are some of the most important issues for MBs operation and this aspect is strongly related to the choice of cell immobilization strategies required for use in analytical devices (Lobsiger & Stark, 2019). In fact, suspended cell cultures, used in typical laboratory settings, are not appropriate for this purpose, where mechanical stability and safe handling are crucial without losing biological activities (Shing et al., 2013; Yoetz‐Kopelman et al., 2016). The literature offers some promising advances in techniques for the immobilization of microbial cells, specifically with the aim of assisting the transition from the laboratory to field application. One of the most popular immobilization techniques is the use of hydrogels. Hydrogels consist of cross‐linked hydrophilic polymers that can retain large amounts of water in their 3D networks, a feature that benefits cell growth (Ahmed, 2015). Among the hydrogels available, the commonly used are agarose and alginate gels (Ma et al., 2019). The ratio between the cells and the matrix must be properly optimized. It is important to note that the authors suggested immediate use of the immobilized cells, indicating that they are not particularly suitable for use after long‐term storage. However, the use of complex matrix can overcome this limitation, as reported by Roda, Cevenini, et al. (2011) who used a mixture of agarose, polyvinylpyrrolidone and collagen for cell immobilization. Both cell viability and activity were maintained up to 35 days at 4°C.

Another form of immobilization is lyophilization, otherwise referred to as freeze‐drying. Lyophilization is a low temperature dehydration process, in which water from a frozen sample is vapourized under vacuum without affecting the integrity of the cells (Lobsiger & Stark, 2019). Sensor cells are typically resuspended in a cryoprotectant solution and spotted onto a platform of choice prior to lyophilization (Stocker et al., 2003). The long‐term storage capabilities enabled by using lyophilization as immobilization strategy is an attractive feature as various authors have reported maintenance of cell viability and sensitivity after months and even years of storage (Martin‐Betancor et al., 2017; Prévéral et al., 2017; Siegfried et al., 2012). Lyophilization techniques address some of the long‐term storage issues, however, the preservation of physiologically active microorganisms presents a significant bottleneck in its efficiency. In this regard, the use of inactive microorganisms, for example, spore form, is a possible solution. *S. cerevisiae* and other microorganisms, such as *Bacillus subtilis* or *Clostridium difficile*, can form spores when exposed to nutrient deficient or harsh conditions unfavourable for their growth (Knecht et al., 2011; Wynn et al., 2017). Notably, it was found that the analytical performance of spore‐based sensors did not change significantly after subjecting them to several sporulation‐germination cycles. Hahne et al. (2021) recently reported high storage capability of up to 6 months at room temperature in yeast for acetic acid detection. Aside from long‐term storage capabilities, the use of spores presents an opportunity to deploy MBs in harsh environments (Sangal et al., 2011).

### Cell immobilization

The implementation of a biosensing concept is as important as the molecular design of the sensor strain. In fact, one of the bottleneck of biosensor development is the transition from a laboratory concept, which is often tested with suspended cells in artificial media, to the realization of a solid, reusable sensor assembly, which performs reasonably well in complex media with multiple and often uncharacterized interfering species (Moraskie et al., 2021). The first design choice is whether to use actively growing or non‐growing cells as sensing element. In fact, actively growing cells in a biosensor produces a variable output: if the analyte is a nutrient, the cell growth rate may change depending on the analyte concentration; if the analyte is biologically active (e.g. toxic), it will alter the growth rate of the biosensing element, thus generating a history‐dependent output (Guo et al., 2021). On the other hand, growing cells allow biosensing element to repair itself in case of toxic shocks, thus minimizing external intervention and extending operative life, which is particularly desirable in remote environments (e.g. seafloor) (Lóránt et al., 2019; Olias & Di Lorenzo, 2021). While these considerations apply to all viable cell biosensors, yeast cells may also sporulate under stress or nutrient‐limiting conditions, which changes their output and eventually the biosensor signal (Hahne et al., 2021). Other phenomena like cell aggregation might alter the surface of the sensing strain exposed to the analyte, thus producing a variable output. In light of these issues, non‐growing cells are preferred over growing cells for practical application of biosensor. Non‐growing cells should be immobilized in a controlled way, so to tailor the sensitivity and the response time of the resulting sensor.

The second design choice concerns the immobilization of the sensing strain. The immobilization increases the mechanical and chemical stability of the sensor assembly, increase resistance to transfer and storage, favours intimate contact with the medium and the analyte, and minimize interference from co‐analytes or competing species thus improving the detection limit. The non‐growing, immobilized cells can be reactivated at the time of use through exposure to the liquid medium containing the analyte. As mentioned earlier, only one commercial biosensor based on viable yeast cells has been published (Lobsiger et al., 2019), which is based on modified *S. cerevisiae* cells lyophilized and immobilized in PEG‐PVA hydrogel. However, several other methods for immobilization and preservation of viable non‐growing cells have been reported, mostly under laboratory or simulated operative conditions.

Immobilization can be achieved through biopolymer (e.g. pectin), which are biocompatible and slowly degrade in soil or water (Srikanth et al., 2008). While this strategy maintains cellular activity and has low environmental impact, the long‐term stability is an issue. Latex or other material that prevent desiccation and increase stability over time have been extensively used for several biotechnological applications, including biosensors. Flickinger group has pioneered and later perfectioned this immobilization technique, obtaining nanoporous coating with engineered interface that maintain cell viability over long time and even dry or harsh conditions (Cortez et al., 2017). However, no commercial application is available yet.

Cell immobilization on paper represents a valid solution in low resource settings for qualitative and/or semi‐quantitative analysis. Yeast‐based paper analytical devices (PADs) have been successfully developed by spotting a suspension mixture of cells, alginate and trehalose as protectant and dried on a paper matrix for the qualitative detection of antibiotics (Weaver et al., 2015). Interestingly, yeast Bio PADs were found to remain viable for more than 1 year when stored at 4°C. A co‐culture of the yeasts *Arxula adeninivorans* and *Debaryomyces hansenii* in presence of glucose as carbon source was immobilized in N‐vinylpyrrolidone‐modified poly(vinyl alcohol) to prepare a BOD biosensor (Yudina et al., 2015). Miller et al. (2020) developed a paper test strip biosensor based on a genetically modified strain of *S. cerevisiae* to detect the antibiotic doxycycline in both human urine and raw bovine serum. The yeast cells were immobilized in a mixture of sodium alginate and trehalose. While this immobilization strategy is suitable only for short‐term applications, it is low‐cost and can be applied in limited technology settings (Miller et al., 2020). *S. cerevisiae* yeast cells have also been immobilized on carbon nanotubes through surface interactions in microbial fuel cells (Christwardana & Kwon, 2017). While this strategy has not been applied to biosensor design, it can be easily used to this purpose.

In a recent study, genetically modified *S. cerevisiae* non‐proliferating cells were immobilized in agarose with carbon source and then placed in a microfluidic device for detection of the drug diclofenac in wastewater (Schirmer et al., 2018). While a follow‐up study has been published (Schirmer et al., 2019), it is unclear if a prototype was later developed. The advantage of using this kind of device is that the cells are safely enclosed within the matrix and the chambers, avoiding signal loss and misinterpretation of the analyte concentration. While the cell immobilization protocols help increasing the biomass concentration and stability, which reflect positively on the biosensor performance, they conversely increase the complexity and the cost of biosensor assembly. Furthermore, the material used for immobilization must be biodegradable if the biosensor is designed for environmental applications.

Self‐immobilization of yeast cells in biofilms offer an interesting alternative to controlled physicochemical immobilization, particularly when the signal is transduced through a redox chain in electrochemical sensors. Biofilms are micro‐structured microbial communities, in which cells are enclosed in microbially produced extracellular matrix, termed extracellular polymeric substance (EPS). EPS confer chemical and mechanical resistance to biofilms, thus extending application of yeast‐based biosensors to high flow rate environment (e.g. wastewater and drinking water pipes), and contaminated water, sediments and soil, where biofilm self‐heals after exposure to contaminants, in principle restoring the sensor performance. Mixed MBs offer a further advantage as the ecological interactions between different species in the community make them more resilient to chemical shocks and more stable in long‐term applications. On the other hand, the presence of EPS delays the diffusion of the analyte in the biofilm, resulting in a transient response (Flemming et al., 2016). Therefore, biofilm‐based MBs cannot be used where rapid response is a priority. Furthermore, biofilm structure is not repeatable, neither in term of physical structure (e.g. thickness, coverage and cluster size), nor in terms of composition (e.g. chemical gradients and co‐localization of different microbial species), which affect the results reproducibility in real‐world applications. Hybrid MBs, in which natural biofilm formation is promoted through cross‐linking, the addition of nanoparticles has been reported (Srikanth et al., 2008). Hybrid or combined immobilization strategies may increase reproducibility of biofilm‐based biosensors. To the best of our knowledge, there are no commercially available biofilm‐based MBs.

### Analytical performance

The assessment of the accuracy of a MB still relies on its comparison with the results of well‐established and highly accurate analytical methods, such as liquid chromatography, mass spectrometry (LC–MS), gas chromatography, mass spectrometry (GC–MS), inductively coupled plasma mass spectrometry (ICP–MS) and other techniques. As accurate as these methods may be, the corresponding instrumentation is costly and require high technical expertise for utilization. On the other hand, selectivity, sensitivity, time of response and limit of detection of MBs is still under question. Selectivity and sensitivity, which in turn determine the time of response and the dynamic range, can be addressed by improving both the sensor strain and the transducer. Currently, methods aimed at manipulating genetic circuitries to improve or alter the activity of biomolecules have given investigators the opportunity to enhance the analytical performance of engineered microbial sensor strains. Directed evolution, a cyclic process mimicking natural selection and aimed at identifying functional biomolecules variants by genetic diversification tools and screening, is one of such technique (Packer & Liu, 2015). Throughout the years, numerous fluorescent proteins have been developed with altered wild‐type genetic makeup to increase their brightness and even modify their excitation and emission wavelengths (Rodriguez et al., 2017). It has been reported that small differences in cell viability between analytical runs can result in signal fluctuations, affecting read‐out reproducibility and detection limits. To address this, investigators have largely relied on the use of optical density measurements and cell counting techniques to determine the quantity of cells in an analytical run. However, these techniques provide no information on the quantity of viable cells that produce a signal and therefore are not the best methods to ensure reproducibility of the measurements. Other approaches, such as the duplex reporter methodologies, have been explored to improve the analytical reproducibility of the sensors. Mirasoli et al. (2002) reported the introduction of a second constitutive reporter gene acting as internal reference for correction of nonspecific response. In the context of variable experimental conditions, the analyte concentration values were corrected by considering the expression of the second reporter gene, providing an internal baseline referred to cell viability and metabolic activity.

Another challenge is the growing interest in engineering sensor strains for use as multiplex assays, in which various analytes can be detected from a single analytical run. This could be achieved by engineering cells with multiple differentiable reporters, each under the control of distinct promoters specific to each analyte of interest. For this purpose, both bacterial and yeast cells have been used. Yoon et al. (2016) reported a multiplex MB for the simultaneous detection of Cd and arsenic in contaminated soils. *Esherichia coli* cells were transformed with two sets of plasmids, one for the detection of arsenic via expression of a red fluorescent protein and another for arsenic detection via GFP expression. Similarly, *S. cerevisiae* cells have been genetically modified with different fluorescent reporters for simultaneous detection of estrogenic or androgenic activities in wastewater samples via high‐performance thin‐layer chromatography (HPTLC) coupled to an optical based bioassay (Moscovici et al., 2020).

Finally, MBs can vary greatly in their time of response. In inducible MBs, microbial cells must first detect a bioavailable analyte and subsequently produce a quantifiable signal. Many factors can play a role that will affect the response time of an inducible sensor strain. This includes the permeability of the cells to a given analyte, the diffusion rate of an analyte through the sample matrix, the complexity of genetic circuitries employed, the strength of the underlying molecular recognition events, the metabolic rate of the microbial cell and the type of reporter molecule employed. Efforts aimed at improving the analytical parameters of a sensor strain, such as incorporating additional regulatory components which could result in lengthening the time of response due to increased metabolic load. In certain cases, time of response can be quite rapid. For example, certain nonspecific and specific MBs can produce result within 1–15 min (Reyes et al., 2020). However, time of response and detection limits are intimately related. In many cases the time of response needs to be lengthened to allow for improved detection limits via increased production of the reporter protein, yielding higher signal‐to‐noise ratios. Thus, the trade‐off between time of response and detection limits allows for tailoring of these parameters to the needs of a given MB application.

### Technical aspects

Apart from the bio‐recognition element, the role of the transduction mechanism is crucial in the analytical performance of MBs and their transition to prototype stage. In this regard, ion sensitive field effect transistors (ISFETs) have several advantages over conventional transducers due to their simple and low‐cost design (Goda & Miyahara, 2013; Kaisti et al., 2016; Park et al., 2017; Prodromakis et al., 2011; Sarangadharan et al., 2018; Tarasov et al., 2016). As the channel region is isolated from the ionic solution, ion‐induced instabilities of sensors such as hysteresis, drift and dissolution of channel materials are prevented, thus extending the durability of the biosensor device (Choi et al., 2012; Fernandes et al., 2010; Guan et al., 2014; Zhou et al., 2014). Field effect transducers (FETs) are reusable, while the sensing parts are disposable, which simplifies the device fabrication and reduces the cost (Park et al., 2017; Prodromakis et al., 2011; Tarasov et al., 2016).

Another challenge that biosensor face is time of response, and it is related to mass transport to transducer (Kelley et al., 2014). The most common method for extending the sensing interface involves the use of magnetic nanoparticles. As dispersing the nanoparticles throughout the sample reduces the distance over which species must diffuse, the response time is thus decreased. This approach switches the sensing paradigm from making the analyte find the sensor to making the sensor find the analyte (Krishnan et al., 2011). For cost‐effectiveness, researchers consider biosensor miniaturization for simple, affordable, and efficient detection and quantification of analytes. Microfluidic platform‐assisted miniaturized biosensing systems show promises in this regard. A miniaturized biosensor will also ease the integration, automation, multiplex detection and transmission of data for use in areas where well‐trained personnel may not be available (Liu et al., 2020; Routray et al., 2019). Nevertheless, further research is needed to enhance the transduction mechanisms, which in turn can help to improve miniaturizing MBs technologies for field applications.

Notably, most of the yeast‐based biosensors developed in the last years are based on an optical transduction mechanism (Table 1), where fluorescence, luminescence or colour are the output signals. Nevertheless, in view of real applications, the implementation of yeast biosensors based on amperometric detection may be taken into consideration given that amperometric biosensors present some advantages in terms of sensitivity, cost of equipment and integration into portable devices (Hassan et al., 2021). For amperometric biosensors, the monitoring process implies measurement of changes in electric current, due to specific cellular reactions and use of specific electrodes (Figure 1). In this frame, yeast capacity to produce protons and electrons from catabolic processes is directly correlated to the biosensor activity (Hassan et al., 2021). Amperometric biosensors are not without their own challenges. Different substrates oxidation processes, aerobic or anaerobic conditions and cellular growth phases have different impact on the electron transfer rate and consequently current output (Hubenova & Mitov, 2015; Mao & Verwoerd, 2013). Another important aspect affecting the electric signal is the cell adhesion to the working electrode which in turn is influenced by the electrode material. Electrode surface should be chemically inert and easy to produce. A wide range of materials have been used such as carbon paper, cloth, graphite foil, rods, metal or metal nanoparticles to modify working electrodes. Among them, graphite has been used for most proof‐of‐concept studies, due to its chemical inertness and ease of production. Furthermore, graphite is biocompatible and can be easily modified to increase the concentration of positively charge functional groups on the surface, which promote cell attachment and biofilm formation.

## FROM LABORATORY SETTINGS TO REAL WORLD APPLICATIONS

**FIGURE 2:**
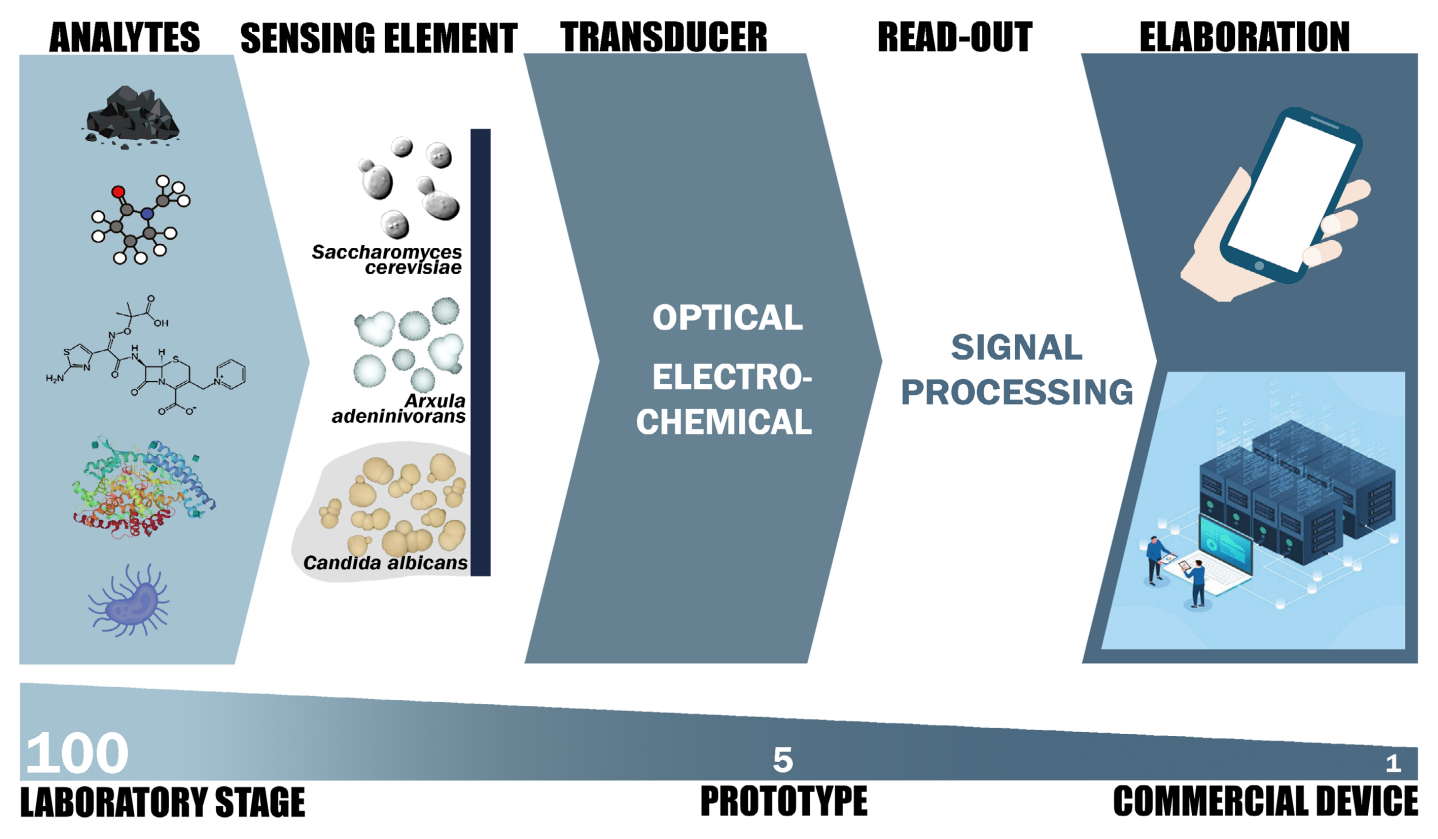
Yeast‐based biosensors: From laboratory stage to commercial device. Different yeast species, including *Saccharomyces cerevisiae, Arxula adeninivorans and Candida albicans*, can function as sensing elements for a variety of analytes. The transduction mechanisms can be essentially optical or electrochemical with relative specific read‐out. The elaboration of the signal processing can be adaptable to economical portable devices and/or Internet of Things systems. Approximately, one hundred yeast biosensors have been developed at laboratory stage, while the prototypes are very limited, about five. Apparently, only one yeast biosensor commercial device is available

Several yeast biosensors have been developed at a laboratory stage for detection of a variety of compounds but moving from research towards practical monitoring is still challenging. The approximate number of prototypes developed from about 100 laboratory stage yeast biosensors is 5 and possibly one of them reached the commercial stage (Figure 2).

**FIGURE 3:**
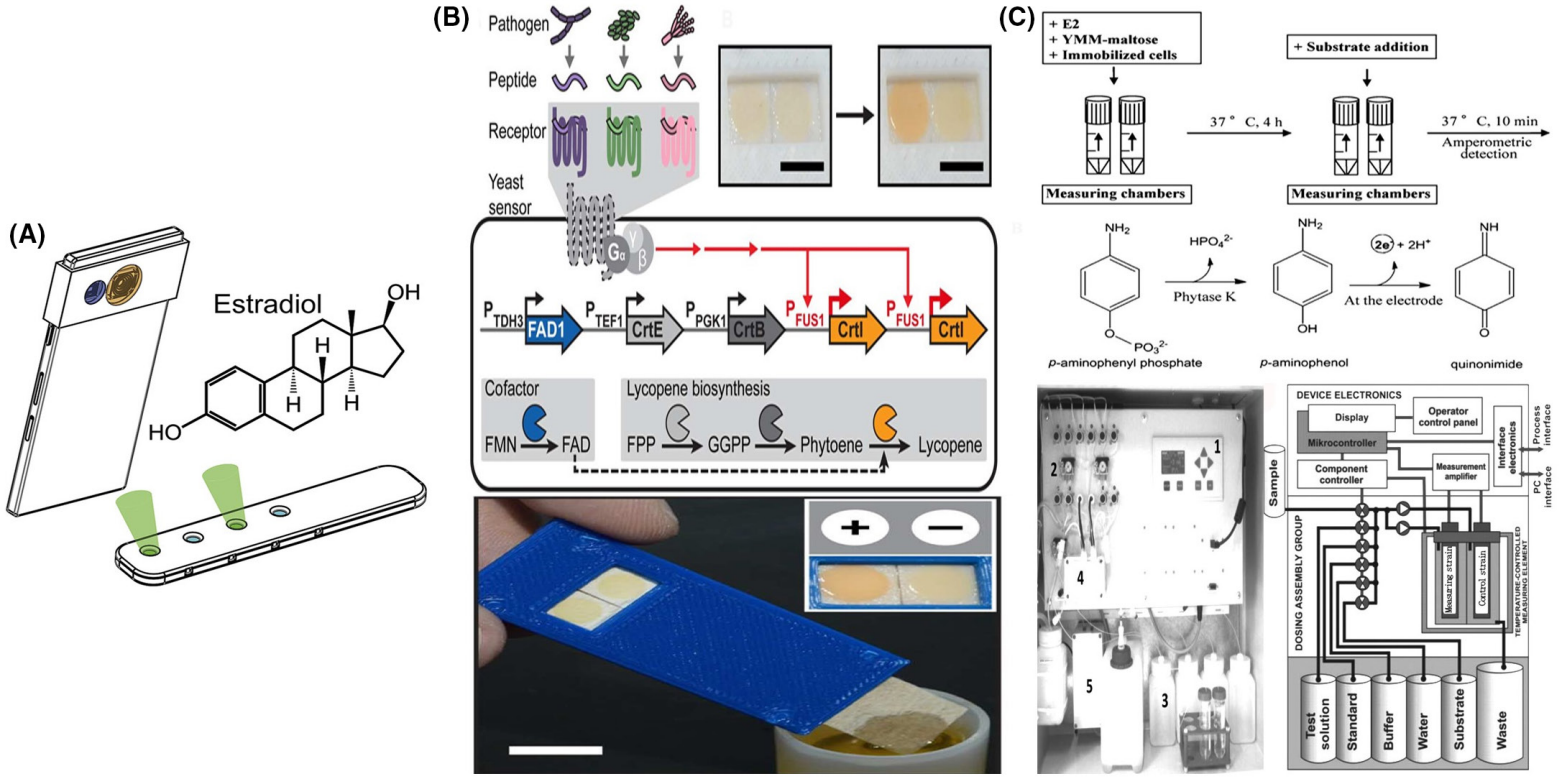
Yeast‐based biosensors prototypes: Working concepts and devices. (A) Yestrosens, a field‐portable and storable *S. cerevisiae* biosensor device for the detection of endocrine‐disrupting chemicals (B) Engineered *S. cerevisiae* cells for on‐site pathogen surveillance adapted to develop a rapid dipstick test which produces either a yes/no response by visual inspection or a quantitative measure by pixel colour analysis (C) EstraMonitor, based on immobilized recombinant *A. adeninivorans* cells contained in measuring chambers for electro‐chemical detection of total estrogenic activity. Selected images are reproduced with permission from the references Lobsiger et al., 2019; Ostrov et al., 2017; Pham et al., 2012

Three prototype models are reported in Table 1 and Figure 3. The first one is from the work by Lobsiger et al. (2019) describing Yestrosens, a field‐portable and storable *S. cerevisiae* biosensor device for the detection of endocrine‐disrupting chemicals. Its design and construction address many shortcomings reported above: cells are lyophilized in a polymeric matrix enabling long term storage and limiting the handling steps; cell cultivation, sensor function and signal detection are integrated within a stimulus‐responsive material; signal readout and quantification are conducted with a nowadays omnipresent electronic device that is, smartphone; the biosensor response include an incubation period of 48 h and a 5 h reaction with the inducer. The second prototype, proposed by Ostrov et al. (2017), has been developed for on‐site pathogen surveillance. In this case, to meet the needs of on‐site detection, the authors engineered *S. cerevisiae* cells by introducing a lycopene production pathway to obtain a robust readout based on this pigment that is easily visible to the naked eye. With this strategy, they developed a rapid dipstick test which produces either a yes/no response by visual inspection or a quantitative measure by pixel colour analysis. The assembled dipsticks maintained their functionality even after long‐term storage. The third prototype, developed by Pham et al. (2012) employed immobilized cells for semi‐continuous detection of estrogenic activity in untreated wastewater samples. While the dipstick reported by Ostrov et al. (2017) does not allow for continuous monitoring of the pollutant, it presents simple immobilization protocol and specificity of the detection mechanism. However, the sensitivity and specificity of the electrochemical biosensor of Pham et al. (2012), even in the presence of high salinity, are sufficient for industrial applications. In fact, the Estramonitor is commercialized by Quodata, Germany (Hettwer et al., 2018).

Some examples of yeast‐based electrochemical biosensors that have been developed, mostly in *S. cerevisiae*: for glucose detection, taking advantage of the yeast cell surface technology (Wang et al., 2015); for blood lead detection, by using yeast cells cross‐linked to Co_3_O_4_/Au composite (Nie et al., 2019); for in situ monitoring of dissolved oxygen levels in environmental waters, with yeast and glucose substrates acting as biocatalyst and fuel, respectively (Christwardana et al., 2021). A microbial consortium, based on bacteria and yeast, has been used to develop amperometric multimetal biosensor (Gao et al., 2016). An interesting electrochemical sensor with *A. adeninivorans* immobilized cells for monitoring of 17β‐Estradiol in environmental samples was reported by Pham et al. (2013). The yeast cells were immobilized in polyvinyl alcohol (PVA) using a genetically modified Lentikat® protocol. The strain used co‐express the *human estrogen receptor α (hERα)* gene and the inducible *phytase* (*phyK*) reporter gene under the control of a promoter containing estrogen response elements (EREs). The authors demonstrated the ability of the sensor to detect estrogenic effects in environmental samples, without the need for sample pre‐treatment, in a semi‐continuous monitoring platform called EstraMonitor (Pham et al., 2012, 2013). Recently, Olaifa et al. (2021) proposed a bioelectrochemical sensor for electroanalysis of *Candida albicans*. In this work, thin biofilm of *Candida albicans* were grown on graphite electrode maintained at oxidative electrochemical potential. In the absence of oxygen as terminal electron acceptor, and in the presence of low concentration of potassium ferricyanide (III) as redox mediator, the viable cells in the biofilm use the poised electrode as electron acceptor, producing a strong baseline current output. When the samples were spiked with antimicrobial and antifungal compounds, such as fluconazole or amphotericin B, the current output decreased proportionally to the concentration of the bioactive molecule tested. These biofilm‐based sensors are particularly interesting because cell immobilization is not required, which simplifies the sensing element design and reduce the cost. However, the long‐term stability under challenging environmental conditions must be assessed.

## CONCLUSIONS AND FUTURE PERSPECTIVES

Basic research, new technologies and deep knowledge of the literature can surely contribute to identifying the technical limitations delaying yeast biosensors applications. The procedures used to integrate the microorganisms and the transducer could help to improve biosensors stability and robustness. Some cell immobilization techniques such as adsorption, encapsulation or covalent binding using different matrices have been already experimented in *S. cerevisiae* (Ponamoreva et al., 2015; Vopálenská et al., 2015; Zaitseva et al., 2017). Also, the ability of certain bacteria and yeast to survive in a transient dormant state (spore) represents an opportunity to obtain long‐term storage MBs (Moraskie et al., 2021). The progress in synthetic and predictive biology, nanotechnology and microfluidics is paving the way for the development of more competitive biosensors. Most of the microbial and yeast biosensors are based on genetic modifications which represent an advantage in terms of specificity and selectivity, although plasmid loss or changes in plasmid copy number may represent caveats in the detection. In this frame, alternative approaches to the introduction of exogenous genetic material can be pursued. For example, by opting for selective modifications in the cellular genome, such as the application of directed evolution technology. Also, taking advantage of intrinsic genetic and/or biochemical properties of the microbial strains, both laboratory strains and indigenous isolates, may be a possible strategy. This latter choice could also enhance the interest in microbial and fungal natural selection and biodiversity, allowing the identification of desired species‐specific properties and avoiding the legislation drawbacks due to the application of genetically modified organisms. In any case, when heterologous expression in microbial systems cannot be avoided, it is preferable to use selective experimental conditions or to opt for exogenous DNA integration into the host genome to increase long‐term stability of the sensor. The biosensor sensitivity can also be improved by integrating co‐expressed genes, encoding the target analyte receptor and a specific intracellular inducible enzyme.

Future research efforts should be focused on how these MBs can be optimized into high‐throughput and economical portable devices that can be deployed for on‐site real time monitoring, especially in agriculture and environmental settings. In this review, we ascertain that most of the developed yeast biosensors are optical, while implementation of electrochemical biosensors may deserve some attention, for their simple equipment, high sensitivity and lower costs. The implementation of electrochemical biosensors based on yeast cells might be a promising solution for on‐site applications. In this regard, the yeast electrochemical capacities can be exploited and adapted to biosensors technology.

## AUTHOR CONTRIBUTIONS

**Ehtisham Wahid:** Conceptualization (equal); writing – original draft (lead); writing – review and editing (supporting). **Ohiemi Benjamin Ocheja:** Conceptualization (equal); writing – original draft (lead); writing – review and editing (supporting). **Enrico Marsili:** Conceptualization (equal); writing – review and editing (lead). **Cataldo Guaragnella:** Conceptualization (equal); funding acquisition (lead); project administration (lead); supervision (supporting); writing – review and editing (supporting). **Nicoletta Guaragnella:** Conceptualization (lead); funding acquisition (lead); project administration (lead); supervision (lead); writing – review and editing (lead).

## CONFLICT OF INTEREST

The authors have no conflict of interest to declare.
